# Detection of multiple signet ring cell carcinomas using texture and color enhancement imaging led to a diagnosis of hereditary diffuse gastric cancer

**DOI:** 10.1002/deo2.70071

**Published:** 2025-01-31

**Authors:** Jumpei Yamamoto, Akira Dobashi, Sei Adachi, Yuta Takano, Kenji Takeshita, Misayo Miyake, Masami Iwamoto, Shintaro Tsukinaga, Naoto Takahashi, Kazuki Sumiyama

**Affiliations:** ^1^ Department of Endoscopy The Jikei University Kashiwa Hospital Chiba Japan; ^2^ Department of Surgery The Jikei University Kashiwa Hospital Chiba Japan; ^3^ Department of Pathology The Jikei University Kashiwa Hospital Chiba Japan; ^4^ Division of Pathology Cancer Institute Hospital, Japanese Foundation for Cancer Research Tokyo Japan; ^5^ Department of Pathology Dokkyo Medical University Tochigi Japan; ^6^ Department of Endoscopy The Jikei University School of Medicine Tokyo Japan

**Keywords:** gastric cancer, hereditary diffuse gastric cancer, image‐enhanced endoscopy, signet ring cell carcinoma, texture and color enhancement imaging

## Abstract

Hereditary diffuse gastric cancer (HDGC) is an autosomal dominant cancer caused by *CDH1* mutation. HDGC causes multiple signet ring cell carcinomas (SRCCs) throughout the stomach. Few reports exist on the endoscopic findings during screening endoscopy, leading to the diagnosis of HDGC in its early stages. Recently, a new image‐enhancement endoscopy technique, texture and color enhancement imaging (TXI), has been developed to improve the visibility of early gastric cancer. To the best of our knowledge, the use of TXI leading to HDGC diagnosis has not been reported. In this report, TXI contributed to the diagnosis of HDGC, and the patient was treated with total gastrectomy. A 27‐year‐old woman with a family history of gastric cancer underwent esophagogastroduodenoscopy, which revealed two pale lesions in the lower body of the stomach. Histological examination of the biopsy specimen revealed SRCC and the patient was referred to our hospital for treatment. Multiple lesions were found in the lower body using TXI, and a targeted biopsy confirmed other SRCCs. We suspected her disease to be HDGC, and the patient underwent a total gastrectomy. Histopathology showed multiple SRCCs (>60), but no lymph node metastases. Genetic testing revealed *CDH1* mutations. The final pathological stage of the tumor was pT1a(m) N0M0 Stage I. TXI may be helpful in detecting multiple SRCCs in patients with HDGC. Endoscopists should be aware of HDGC, and careful investigation of the entire stomach is required for patients with diffuse‐type gastric cancer before treatment.

## INTRODUCTION

Hereditary diffuse gastric cancer (HDGC) is an autosomal dominant cancer syndrome characterized by a high prevalence of diffuse‐type gastric cancer and lobular breast carcinoma. It is primarily caused by the inactivation of germline mutations in the tumor suppressor gene *CDH1*.^1^ The concept of HDGC was first proposed at the International Gastric Cancer Linkage Consortium in 1999.^2^ HDGC has been reported in various ethnic groups, mainly in Europe and the United States; however, only a few reports exist regarding HDGC in Japanese patients.^3^

Recently, image‐enhanced endoscopy, including texture and color enhancement imaging (TXI), has been reported to improve the visibility of gastric cancer (GC).^4^ We encountered a Japanese patient with HDGC and multiple signet ring cell carcinomas (SRCCs), diagnosed based on endoscopic findings using TXI.

## CASE REPORT

**FIGURE 1:**
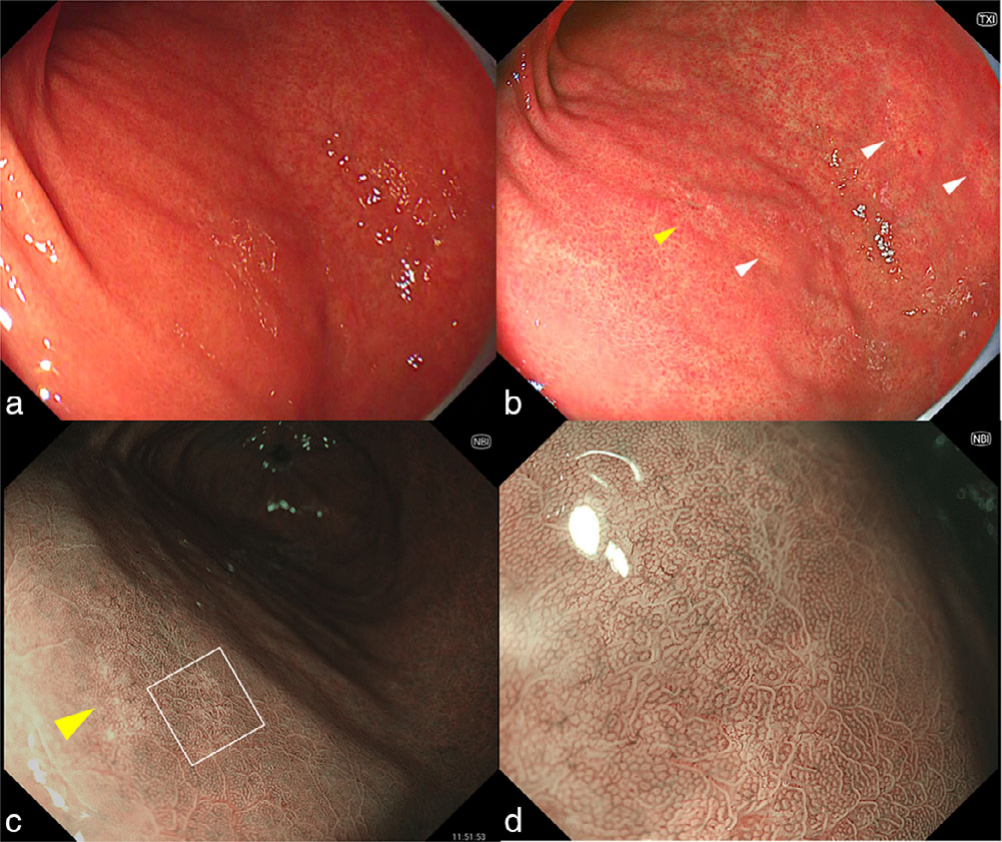
Endoscopic images of hereditary diffuse gastric cancer. (a) Gastric cancers are difficult to identify with white light imaging. (b) Texture and color enhancement imaging reveals multiple pale lesions (yellow and white arrowheads). Close examination with narrow‐band imaging is performed on the lesion (yellow arrowheads). (c) Narrow band imaging identifies the lesion as whitish. A demarcation line is also observed. (d) On magnifying endoscopy with narrow‐band imaging, the microsurface pattern of the lesion appears mostly regular; however, in some areas, the surface appears irregular or absent. Areas that appear absent showed slightly irregular microvasculature.

**FIGURE 2:**
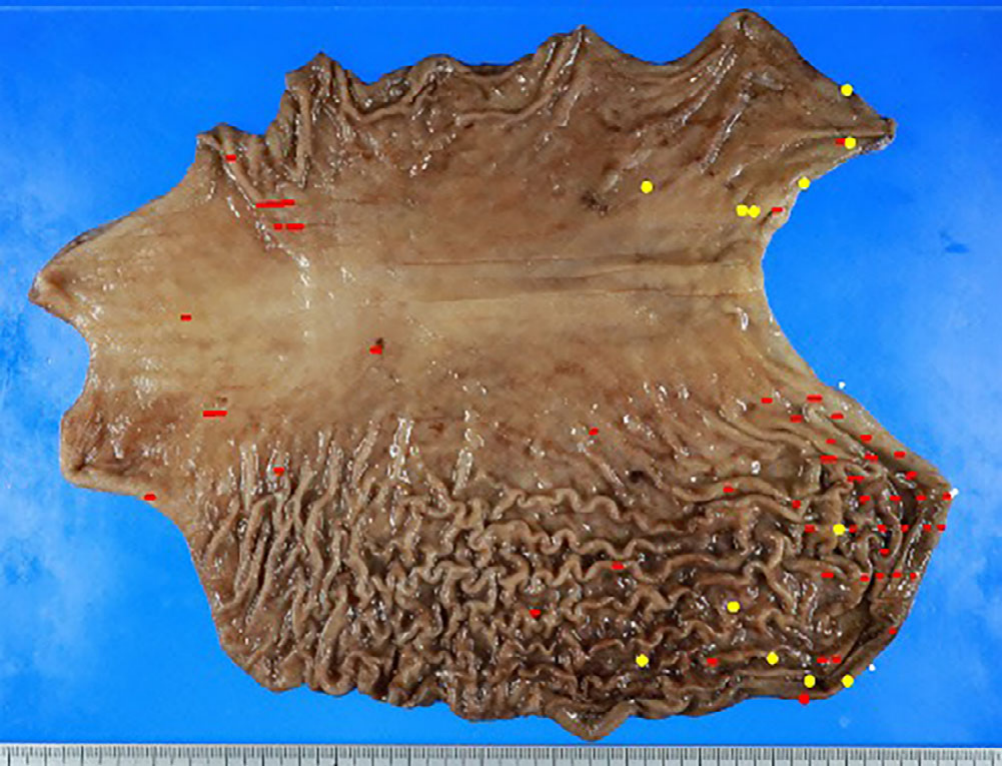
Distribution of the carcinomas in the whole resected specimen. More than 60 multiple signet ring cell carcinoma lesions have been identified. All lesions are confined to the mucosal layer, encompassing the foci of the intramucosal carcinoma (red dots) and carcinoma in situ (yellow dots).

**FIGURE 3:**
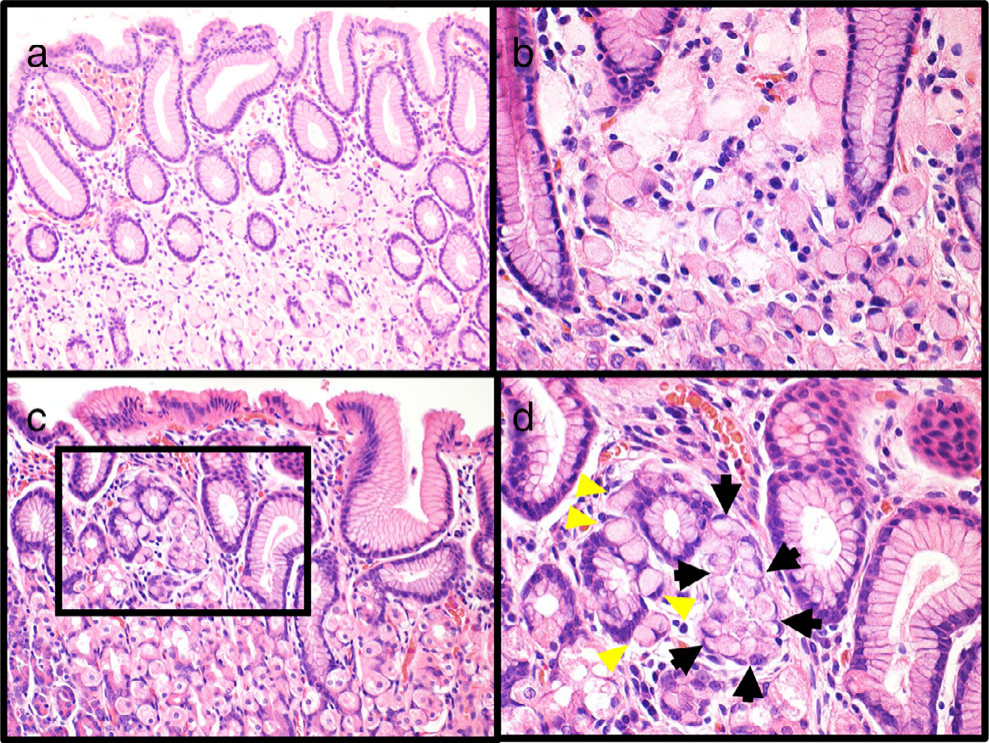
Histopathological features of pT1a and pTis signet ring cell carcinomas (hematoxylin and eosin staining). (a, b) Signet ring cell carcinoma (pT1a) cells are also present in the mucosal layer. Although the carcinoma cells have invaded, the gastric pit density and surface foveolar epithelium are preserved. (c, d) Signet ring cell carcinoma in situ (black arrows) and pagetoid spread of signet ring cells (yellow arrowheads) are observed in the neck zone with preservation of the conventional gastric gland shape.

A 27‐year‐old woman underwent screening esophagogastroduodenoscopy at a clinic after her brother died from scirrhous GC in his 20s, and her father and grandmother were treated for GC (Figure S1). Two pale lesions <5 mm in size were found in the lower body at the lesser curvature, and the biopsy specimens were histologically diagnosed as SRCC. She was referred to our hospital for endoscopic resection. The patient underwent esophagogastroduodenoscopy using a magnifying endoscope (GIF‐XZ1200; Olympus) at our hospital. White light imaging (WLI) revealed a regular arrangement of collecting venules, indicative of *Helicobacter pylori*‐negative stomachs, at the gastric angle. The pale lesions detected in previous examinations were obscured. However, by switching the modality from WLI to TXI mode 1, we identified two pale lesions and observed other pale lesions < 5 mm in the antrum (Figure 1) because the color difference between the lesion and the surrounding normal mucosa was emphasized. The lesion had a demarcation line on narrow‐band imaging (NBI). The microsurface pattern of the lesion appeared mostly regular; however, in some areas, the surface appeared irregular or absent. The areas that appeared absent showed slightly irregular microvasculature. We diagnosed the pale lesions as GC by endoscopic examination. We performed biopsies of the four lesions, and the other SRCCs were diagnosed histologically (Figure S2). Blood antigen tests revealed no *H. pylori* infection. Under these circumstances, we suspected HDGC and strongly suggested total gastrectomy instead of endoscopic resection or distal gastrectomy. Because HDGC is associated with lobular breast cancer, the patient underwent breast ultrasonography. She also underwent colonoscopy, colposcopy, and computed tomography of the chest and abdomen; no malignancies were detected. The patient desired early treatment; therefore, total gastrectomy with lymph node dissection was performed before the genetic findings were obtained. Histological analysis of the entire resected specimen, including 209 slices, confirmed >60 SRCCs (Figure 2). The pathological findings revealed multiple foci of intramucosal SRCCs. The maximum tumor size was 5 mm, located in the lower third of the stomach, and showed pT1a (m) (Figure 3a,b). In pT1a lesions, normal fundic glands are destroyed by SRCC; however, the surface foveolar epithelium is preserved. The lesions in the upper third of the stomach, where we could not identify SRCCs endoscopically before surgery, were < 3 mm in size, and SRCC in situ (pTis) were observed (Figure 3c,d). In pTis, SRCCs were observed in the neck zone with preservation of the conventional shape of the gastric glands. Pagetoid spread of signet ring cells was observed in the upper third of the stomach, and the degree of destruction of normal fundic glands was lower than that in the lower third of the stomach. The pagetoid spread of signet ring cells is a characteristic of HDGC, indicating that our patient had a high risk of HDGC. No lymph node metastasis was observed. The final pathological stage of the tumor was pT1a (m) N0M0 Stage IA. Genetic testing after gastrectomy revealed *CDH1* c.1565+1G > A variant, which led to the diagnosis of HDGC.

## DISCUSSION

GC is one of the most common cancers worldwide, the fifth most common cancer, and the fourth leading cause of cancer‐related deaths in 2020, with 1.1 million new cases and 770,000 GC‐related deaths.^5^ The vast majority of GCs are sporadic; however, it has now been established that 1–3% of GCs worldwide arise because of inherited cancer predisposition syndromes.^6^ The lifetime risk of inherited GC varies substantially between the populations studied but is generally low. In contrast, the risk of GC in patients with *CDH1* germline mutations is reported to be 70% in men, 56% in women, and 42% in women with lobular breast carcinoma by the age of 80 years,^6^ indicating a significantly higher incidence of GC than that of other hereditary diseases. HDGC detected via endoscopy is often advanced, and the 5‐year survival rate of patients with HDGC is very low (<30%). Only three cases have been reported in which endoscopic findings led to the diagnosis of HDGC without prior knowledge of *CDH1* mutation (Table 1).

**TABLE 1:** Cases of hereditary diffuse gastric cancer diagnosed in the early stage during screening endoscopy without prior knowledge of CDH1 genetic mutation.

Case	Author	Year	Race	Age	Sex	Positive family history of gastric cancer	Treatment	Stage (UICC^*^)
1	Yamada^3^	2014	Asian (Japanese)	34	Female	In son, father, and grandfather	Distal gastrectomy with Billroth I reconstruction followed by total gastrectomy	T1aN0M0, stage 1A
2.	Iwaizumi^9^	2020	Asian (Japanese)	24	Male	In mother	Total gastrectomy	N/A
3.	Iwaizumi^9^	2020	Asian (Japanese)	35	Male	In father and father's brother	Distal gastrectomy followed by total gastrectomy	N/A
4.	Namikawa^10^	2021	Asian (Japanese)	34	Male	Positive family history of gastric cancer but not precisely noted	Total gastrectomy	T1aN0M0, stage 1A
5.	Our case	2024	Asian (Japanese)	27	Female	In elder brother, father, and grandmother	Total gastrectomy	T1aN0M0, stage 1A

* Eighth edition of the UICC (Union for International Cancer Control) classification of malignant tumors.

Our patient was from Japan where HDGC is rare. Although HDGC may be difficult to diagnose endoscopically in its early stages, as in our case, we detected multiple SRCCs using TXI. To the best of our knowledge, this is the first report of endoscopic examination under TXI for the diagnosis of HDGC. The TXI is a newly developed image‐enhanced endoscopy that enhances the texture, brightness, and color of endoscopic images obtained using WLI. Futakuchi et al. reported that TXI improved the visibility of gastric neoplasms compared with WLI and that TXI enhanced the visibility of early GC regardless of lesion color, morphological type, location, *H. pylori* infection status, atrophic gastritis, histology, and intestinal metaplasia.^4^ As in our case, SRCCs were difficult to detect using WLI. However, TXI improved the color differences, and multiple small pale lesions were identified.

In the present case, most of the pT1a lesions in the antrum were detected and diagnosed endoscopically. This may be due to the high degree of destruction of the gastric fundic gland by SRCC. Although the surface foveolar epithelium was preserved, the SRCC was densely distributed in the mucosal layer, allowing visualization of the lesion as a pale area on TXI. Furthermore, magnifying endoscopy with NBI revealed that the boundary had a demarcation line. Irregular microsurfaces and microvascular patterns have also been observed, leading to a diagnosis of GC.^7^ Although we detected multiple GCs with TXI in the lower third of the stomach, it is difficult to diagnose all cancers accurately in patients with HDGC. According to Fujita et al., patients with HDGC have the highest density of GCs in the anterior proximal fundus (37%) and cardia/proximal fundus (27%)^8^; however, we could not endoscopically detect GCs in the upper third of the stomach. Based on our histopathological analysis of the entire resected specimen, the GCs in the upper third of the stomach were <3 mm in size. Additionally, GCs in the upper third of the stomach may have been difficult to detect endoscopically because they were mainly pTis, in which the gastric pit density and surface foveolar epithelium were preserved and the destruction of the fundic glands was not obvious.

With the development of endoscopic technologies such as TXI, HDGCs can now be detected at an early stage. Endoscopists should be aware of the disease and endoscopic findings of HDGC. Careful investigation of the entire stomach is required before treatment in patients with diffuse‐type gastric cancer.

## CONFLICT OF INTEREST STATEMENT

All authors had full access to the data and had the final responsibility for the decision to submit for publication. No benefits in any form have been received or will be received from any commercial party directly or indirectly related to the subject of this study. Author Kazuki Sumiyama is the DEN Open's Deputy Editor‐in‐Chief.

## ETHICS STATEMENT

This case report did not include any analysis of human or animal subjects by any of the authors.

## PATIENT CONSENT STATEMENT

The patient provided informed consent for this case report.

## Supporting information




**FIGURE S1** Family pedigree. The black arrows indicate the present case. Individuals with gastric cancer are shaded black. GC: gastric cancer. DGC: Diffuse type gastric cancer.

**FIGURE S2** Endoscopic image of gastric cancer and neighboring area. A. Under white light imaging, a pale lesion in the lesser curvature of the body was obscured. B. In texture and color enhancement, color is emphasized, and the lesion is easier to detect as a whitish area. A biopsy was performed, and the lesion was diagnosed as signet ring cell carcinoma.

## References

[1] Blair RV , McLeod M , Carneiro F *et al*. Hereditary diffuse gastric cancer: Updated clinical practice guidelines. Lancet Oncol 2020; 21: e386–e397. 32758476 10.1016/S1470-2045(20)30219-9 PMC7116190

[2] Caldas C , Carneiro F , Lynch HT *et al*. Familial gastric cancer: Overview and guidelines for management. J Med Genet 1999; 36: 873–880. 10593993 PMC1734270

[3] Yamada M , Fukagawa T , Nakajima T *et al*. Hereditary diffuse gastric cancer in a Japanese family with a large deletion involving CDH1. Gastric Cancer 2014; 17: 750–756. 24037103 10.1007/s10120-013-0298-y

[4] Futakuchi T , Dobashi A , Horiuchi H *et al*. Texture and color enhancement imaging improves the visibility of gastric neoplasms: Clinical trial with image catalogue assessment using conventional and newly developed endoscopes. BMC Gastroenterology 2023; 23: 389 37957560 10.1186/s12876-023-03030-9 PMC10644425

[5] Morgan E , Arnold M , Camargo MC *et al*. The current and future incidence and mortality of gastric cancer in 185 countries, 2020−40: A population‐based modelling study. EClinicalMedicine 2022; 47: 101404 35497064 10.1016/j.eclinm.2022.101404 PMC9046108

[6] van der Post RS , Vogelaar IP , Carneiro F *et al*. Hereditary diffuse gastric cancer: Updated clinical guidelines with an emphasis on germline *CDH1* mutation carriers. J Med Genet 2015; 52: 361–374. 25979631 10.1136/jmedgenet-2015-103094 PMC4453626

[7] Muto M , Yao K , Kaise M *et al*. Magnifying endoscopy simple diagnostic algorithm for early gastric cancer (MESDA‐G). Dig Endosc 2016; 28: 379–393. 26896760 10.1111/den.12638

[8] Fujita H , Lennerz JKM , Chung DC *et al*. Endoscopic surveillance of patients with hereditary diffuse gastric cancer biopsy recommendations after topographic distribution of cancer foci in a series of 10 *CDH1*‐mutated gastrectomies. Am J Surg Pathol 2012; 36: 1709–1717. 23073328 10.1097/PAS.0b013e31826ca204

[9] Iwaizumi M , Yamada H , Fukue M *et al*. Two independent families with strongly suspected hereditary diffuse gastric cancer based on the probands’ endoscopic findings. Clin J Gastroenterol 2020; 13: 754–758. 32594425 10.1007/s12328-020-01163-y

[10] Namikawa K , Kawachi H , Tsugeno Y *et al*. Detection of multiple intramucosal signet‐ring cell carcinomas by white‐light endoscopy and magnifying endoscopy with narrow‐band imaging in a hereditary diffuse gastric cancer patient with a CDH1 germline mutation. VideoGie 2021; 6: 163–166. 33898891 10.1016/j.vgie.2020.11.020 PMC8058510

