# Supplementary figures and images for: Detection of multiple signet ring cell carcinomas using texture and color enhancement imaging led to a diagnosis of hereditary diffuse gastric cancer
Source: DEN Open. 2025 Jan 31;5(1):e70071. doi: 10.1002/deo2.70071 (PMC11783146; doi:10.1002/deo2.70071)

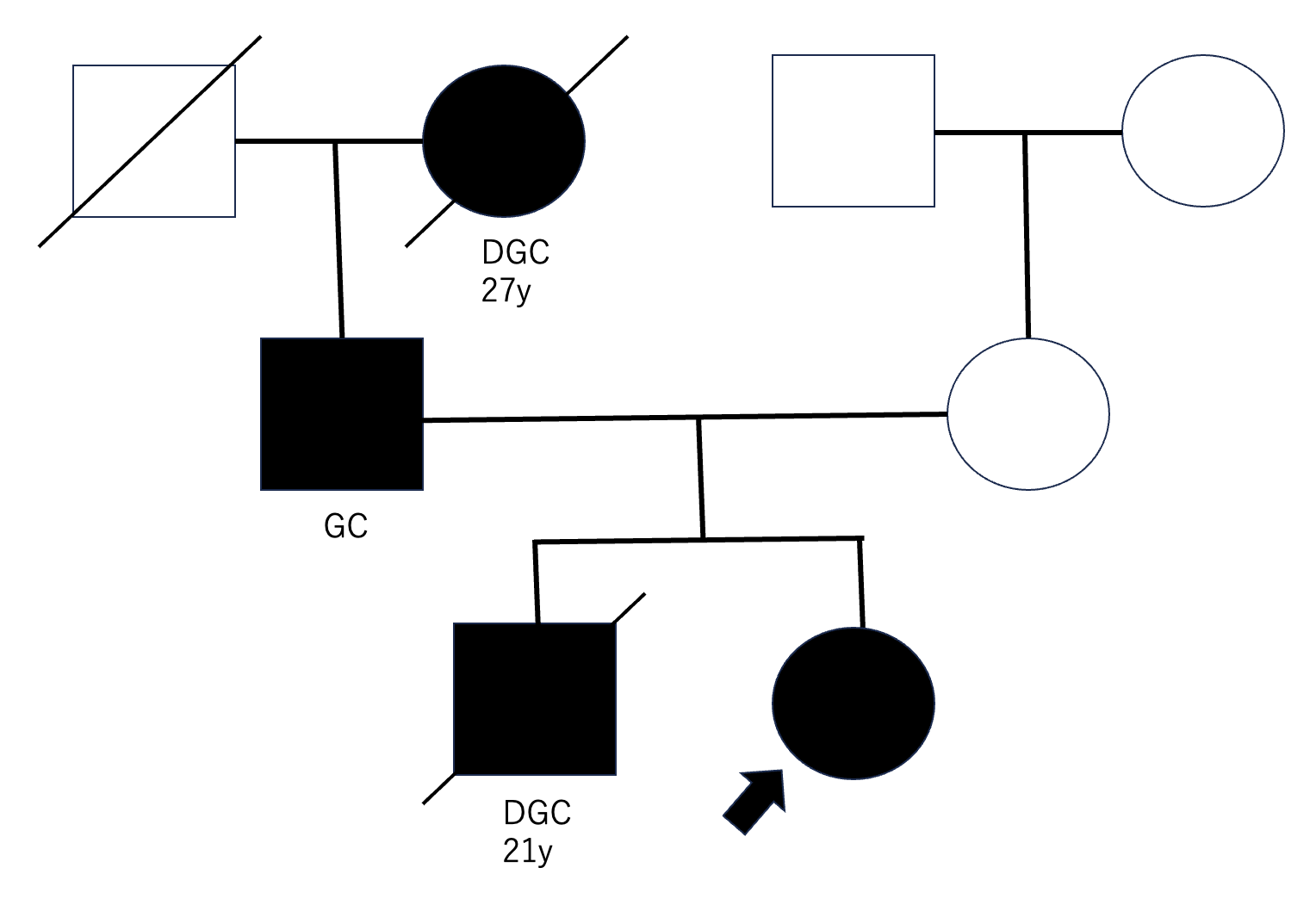

Supplement: Supplementary file 1 — FIGURE S1 Family pedigree. The black arrows indicate the present case. Individuals with gastric cancer are shaded black. GC: gastric cancer. DGC: Diffuse type gastric cancer. [file DEO2-5-e70071-s001.png]

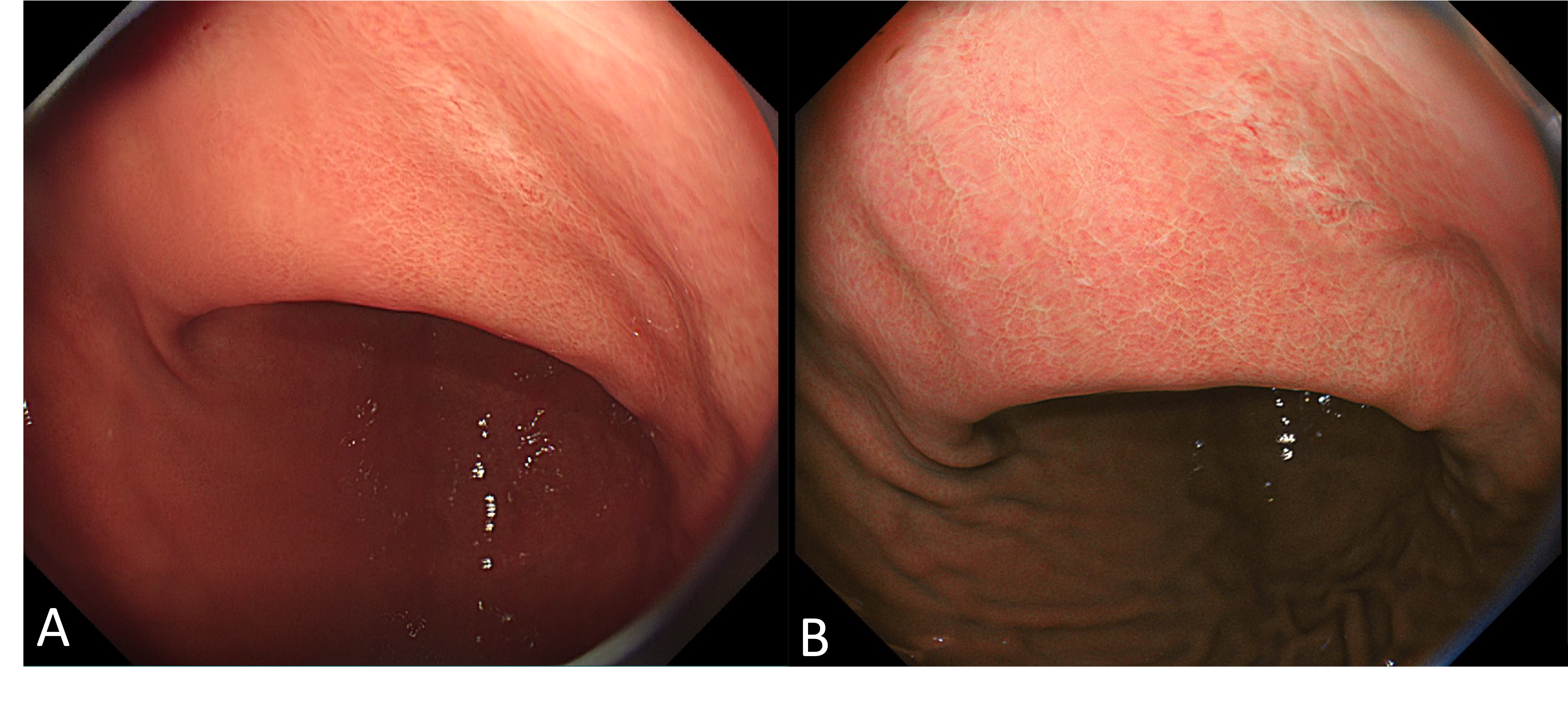

Supplement: Supplementary file 2 — FIGURE S2 Endoscopic image of gastric cancer and neighboring area. A. Under white light imaging, a pale lesion in the lesser curvature of the body was obscured. B. In texture and color enhancement, color is emphasized, and the lesion is easier to detect as a whitish area. A biopsy was performed, and the lesion was diagnosed as signet ring cell carcinoma. [file DEO2-5-e70071-s002.png]
